# Participatory evaluation of chicken health and production constraints in Ethiopia

**DOI:** 10.1016/j.prevetmed.2014.10.014

**Published:** 2015-01-01

**Authors:** Emmanuel Sambo, Judy Bettridge, Tadelle Dessie, Alemayehu Amare, Tadiose Habte, Paul Wigley, Robert M. Christley

**Affiliations:** a The Institute of Infection and Global Health, University of Liverpool, Leahurst Campus, CH64 7TE, United Kingdom; b International Livestock Research Institute, Addis Ababa, Ethiopia; c Ethiopian Institute of Agriculture Research, Debre Zeit Agricultural Research Center, Debre Zeit, Ethiopia; d NIHR Health Protection Research Unit in Emerging and Zoonotic Infections, Liverpool L69 7BE, United Kingdom

**Keywords:** Poultry production constraints, Poultry diseases, Perceived disease risk factors

## Abstract

Chicken production has a major role in the economy of developing countries and backyard production is particularly important to women. Several programmes, in Ethiopia and elsewhere, have attempted to improve chicken production as a means to reduce poverty. A key constraint to chicken production identified by farmers is disease. This study used participatory rural appraisal methods to work with chicken-keepers in order to prioritise chicken diseases, place these within the context of other production constraints, and to explore perceptions of disease risk factors and biosecurity measures.

The study, focused on Debre Zeit, Ethiopia, included 71 poultry keepers (41 backyard and 30 semi-intensive chicken producers). Although women played an important role in backyard production systems, semi-intensive farms were more likely to be controlled by men. Participants identified 9 constraints to production: 7 of 8 groups of backyard producers and 15/31 semi-intensive producers ranked diseases as the most important constraint to chicken production. In contrast to previous reports, farmers in both groups had considerable knowledge of diseases and of factors affecting disease risk. Both groups, but particularly semi-intensive producers, highlighted access to feed as a constraint. Many of the challenges faced by both groups were associated with difficulty accessing agricultural and veterinary inputs and expertise.

Whilst many of the constraints identified by farmers could be viewed as simply technical issues to be overcome, we believe it is important to recognise the social factors underpinning what are, in reality, relatively modest technical challenges. The low involvement of women in semi-intensive production needs to be recognised by poultry development schemes. Provision needs to be made to allow access to inputs for a wide range of business models, particularly for those, such as women, who have limited access to the capital to allow them to make the jump from backyard to semi-intensive producer, and require support to slowly build up a flock into a profitable venture.

## Highlights

• We examine chicken health and production constraints with backyard and semi-intensive producers in Ethiopia.
• Participants identified 9 categories of constraints: the most important overall were disease and access to feed.
• Despite generally detailed knowledge of disease risk factors, biosecurity measures were lacking or ineffective.
• Producers reported difficulty accessing veterinary services, despite their proximity to such expertise.
• Although many of these constraints may be viewed as technical issues to be overcome, our findings highlight underlying social factors that must also be addressed.

## 1 Introduction

Poultry production has a major role in the economy of developing countries, including an important role in poverty alleviation by means of income generation and household food security (FAO, 1997; Gondwe, 2004; Abdelqader et al., 2007; Abubakar et al., 2007). More than half of Ethiopian households both in rural and urban areas keep chickens, although there is considerable variation in the distribution of chicken keeping, with most households in highland areas keeping chickens, and far fewer doing so in lowland pastoral areas (Ayele et al., 2009; Wilson, 2010). Production is characterised by free range backyard or village systems (Sonaiya, 1990a,b; Guèye, 2003) and chicken production is considered an integral part of many families’ livelihoods (Tadelle et al., 2003). Studies across Africa, and in Ethiopia in particular, show women often directly control the income generated from the sale of chickens and chicken products, and that this is sometimes their only source of independent income. Hence, chicken production is important in developing countries where options for income generation for women are limited (Bradley, 1992; Guèye, 1998; Bravo-Baumann, 2000; Pederson et al., 2001; Dessie and Ogle, 2001; Seeberg, 2002; FAO, 2004; Riise et al., 2005; Aklilu et al., 2007; Halima et al., 2007; Wilson, 2010).

The majority (94–99 per cent) of the chicken population in Ethiopia, estimated to be 49 million in 2011 (CSA, 2010/11), are indigenous local breeds (CSA, 2005; Alemu et al., 2008). Chicken production has occurred largely on small farmer holdings, with an average flock size of 4.1 (CACC, 2003; CSA, 2005), limited capital investment and few inputs (Sonaiya, 1990a; Guèye and Bessei, 1996; Guèye, 1998; FAO, 2004; Alemu et al., 2008). Wilson (2010) provides an overview of chicken production in Ethiopia.

In 1996, the Ethiopian Ministry of Agriculture developed a poultry extension package for rural farmers which involved training a member of the household in various aspects of poultry management, and providing a nucleus flock of Rhode Island Red chickens (Dessie and Jobre, 2004). The programme was not a great success, as the exotic birds showed a poor tolerance to the local conditions, and farmers have complained that this distribution of exotic cocks, pullets and fertile eggs has negatively impacted on the local poultry's brooding ability and adaptation to low-input feeding systems (Dinka et al., 2010). However, a report by Pagani and Wossene (2008) described the poultry multiplication and distribution centres as an unqualified success, and there is evidence that they have helped chicken production in urban and peri-urban areas to become a profitable venture over the last 15–20 years, with more families keeping small to medium-size flocks (approximately 50–1000 birds) under semi-intensive management (FAO, 2008). Entrepreneurs are also investing in the industry with larger flocks of exotic breeds kept under intensive management (FAO, 2008; Wolde et al., 2011). Although these commercial farms have been set up in order to meet the increased demand for poultry products from an emerging middle-class urban sector, most Ethiopians still exhibit a strong preference for indigenous poultry products as meat and eggs from exotic breeds are perceived to have poorer taste (Dana et al., 2010). Therefore the traditional poultry sector still fulfils a viable role producing birds for the domestic market.

A number of challenges and obstacles (which we here call ‘constraints’) limiting the success and profitability of both backyard and semi-intensive production have been identified, including infectious diseases, low input of veterinary services, poor housing, poor biosecurity, predators and, the quality and cost of feed (Demeke, 1996; Wossene, 2006; Woldemariam and Wossene, 2007; Alemu et al., 2008; Ayele et al., 2009; Wolde et al., 2011; Mazengia, 2012). However, these studies often focus on one or a few constraints and have not assessed the knowledge and beliefs of the chicken producers themselves.

Livestock keepers are a rich source of information about breeds and production systems and also important diseases which affect their animals (Catley and Mariner, 2001; Adesehinwa et al., 2003). Utilising this information, called ‘existing veterinary knowledge’ (Mariner and Paskin, 2000), through a participatory approach that allows open and flexible discussion, may lead to better delivery of veterinary services which are in tune with the priorities of the community.

The aim of this study was to investigate, using participatory research methods, the constraints facing both backyard and semi-intensive chicken farmers in and around Debre Zeit, Ethiopia; with a particular focus on the disease problems, farmers’ perceptions regarding disease risk factors and the biosecurity measures in place on these farms. Our goal is to identify key issues to be addressed in order to facilitate the role of chicken production in Ethiopian livelihoods. This region was deliberately selected for study because it is the focus of chicken production and services in Ethiopia. Hence, constraints identified here are likely to be felt more acutely elsewhere in Ethiopia (and, indeed, in much of Africa), where they may be compounded by additional infrastructural limitations affecting communication and transportation. Thus, our results highlight constraints that will need to be overcome even following improvement in these infrastructural limitations.

## 2 Materials and methods

### 2.1 Ethical approval

This study (including the process of obtaining informed consent) was approved by the University of Liverpool Veterinary Research Ethics Committee (reference VREC33). Participants were provided with verbal information to inform them of the purpose of the study, that participation was entirely voluntary, that they were free to leave the study at any time and that all data would be kept securely. Verbal informed consent was obtained prior to collection of data. Verbal information and verbal informed consent was deemed appropriate due to the expectation of relatively low literacy levels among participants. Consent was documented for each participant by a tick box on the information sheet that was read to each potential participant and which was ticked in the presence of the participant.

### 2.2 Study setting

Debre Zeit (also called Bishoftu) is a city of approximately 170,000 people 50 km south of Addis Ababa, the capital of Ethiopia, and is a focus of intensive and semi-intensive chicken production in Ethiopia (FAO, 2008; USAID, 2010). Debre Zeit is also the location of the University of Addis Ababa College of Veterinary Medicine and Agriculture, the National Veterinary Institute, the Pan-Africa Veterinary Vaccine Centre, and an Ethiopian Institute of Agricultural Research centre focused on chicken production. Hence, this area has greater potential for access to veterinary and production expertise and materials to support chicken production than anywhere else in the country.

In addition to traditional healers, who use ethnoveterinary knowledge to treat and prevent disease, Ethiopia has three categories of formal animal health workers (in decreasing order of level of training): veterinarians, animal health assistants and animal health technicians (Admassu, 2003). Animal health services are predominantly within the public sector; in 2003, 90% of veterinarians worked in the government services. The Ministry of Agriculture operates regional clinics (staffed by veterinarians, animal health assistants and animal health technicians) and health posts staffed by animal health technicians. Despite this, these facilities are often understaffed, and effective animal health services are not available to the majority of livestock owners (Admassu, 2003). Provision of private sector animal health services is increasing, particularly in the capital city, Addis Ababa, and other major urban centres, including Debre Zeit. However, private sector services have tended to concentrate on pharmaceutical importation and sales, and to a lesser extent on provision of clinical services (Admassu, 2003).

### 2.3 Study participants

This study included two types of chicken-keepers; backyard producers and semi-intensive producers. Backyard producers were defined as those who kept their flock predominantly under a free-range scavenging system, whereas semi-intensive producers were defined as those who predominantly confined their flock in purpose-built housing. Farmers were identified through local Ministry of Agriculture development agents, who were asked to invite participants of both genders and of various ages. Eight volunteer focus group interviews were conducted with backyard producers in 4 villages in the area of Debre Zeit, Ethiopia: Diree; Kaltika; Ude; and Sirba.

The villages were selected on the basis of access and willingness to participate. Each focus group (two in each village) was comprised of 5–6 participants and included a mix of genders and age groups and were held in Farmer Training Centres in each village. Individual interviews were conducted with semi-intensive poultry farmers in the same area. These interviews were conducted at the farm and included observation of current stock, buildings and biosecurity practices. In addition, interviews were conducted with three local key informants, namely veterinary service providers. Several participatory research tools, including semi-structured interviews, simple ranking, proportional piling and seasonal calendars were used for data collection. Fieldwork for the study occurred between 19th May and 13th June 2012.

### 2.4 Participatory methods

Advocacy visits were made to each community prior to the proposed focus-groups/interviews and the necessary permission obtained from key officials and respondents. Depending on the preference of the participants, either of the two main local languages (Amharic or Afan Oromo) was used for communication through animal health assistants trained in participatory research methods.

Where respondents were asked to identify or list specific items, these were written on cards or represented by pre-prepared picture cards. These cards were used for simple ranking for interviews with semi-intensive producers, with the cards placed in the desired order of importance, or for proportional piling in the focus groups with backyard producers, where counters (usually 100 or 30) were used to show the relative importance placed on an item by piling the counters on the desired item. After each exercise, respondents were given the opportunity to make changes to their ranking or scoring before the final results were recorded. Preliminary results of each exercise were communicated to the respondents and further discussed to gain deeper insights as to why they made their choice(s).

Materials used include counters (beans), flip charts, cards of different colours, permanent markers and various pictures of livestock species kept in Ethiopia. Representative pictures of animals were used where necessary to ease understanding of each exercise. The elicitation process involved semi-structured interviews with open-ended questions used to encourage discussion. The respondents were allowed to freely express themselves on issues raised with minimal interruptions. Focus group discussions and interviews lasted approximately 1 h.

A single checklist was used to guide and standardise discussions in the focus groups with backyard chicken keepers and the interviews with semi-intensive producers. The aim of the discussions was to investigate the role of chickens within peoples’ livelihood strategies and to identify constraints to chicken production. In order to facilitate discussion, the checklist began with more general topics before introducing more specific areas. Discussion began with introduction of the study team and explanation the purpose of the visit. There was the opportunity for the group to raise questions regarding the research before informed consent was obtained from participants. The discussion then explored the range of livelihood activities undertaken by the participants, focusing on livestock activities. The respondents then identified the important types of livestock; initially, these were listed then compared, using proportional piling (backyard producers) or simple ranking (semi-intensive producers), on the basis of their perceived economic importance. Subsequently poultry were singled out, and information about flock size and management activities was elicited. Participants were then asked to discuss the challenges and obstacles (i.e. constraints) they faced in poultry production and a list of these constraints was produced. These were then compared using proportional piling (backyard producers) or ranking (semi-intensive producers). From this list, disease was singled out for further discussion. Initially participants were asked to identify diseases. The clinical presentation of each identified disease was discussed and then the listed diseases were compared in terms of mortality, using proportional piling (backyard producers) or ranking (semi-intensive producers). A seasonal calendar was then constructed for each disease in order to identify times at which the diseases named by the participants occurred.

Discussion then turned to factors thought to affect the risk of disease in the flock. Again, these were listed then compared in terms of perceived importance, using proportional piling (backyard producers) or ranking (semi-intensive producers). Participants were also asked to identify possible biosecurity measures that could be used to reduce disease entry to their flocks: specifically, they were asked to identify all such practices they could, whether or not they were performed. Discussion then turned to whether or not each measure was implemented and, where a measure was not implemented, the reason why was sought. In addition, direct observations of chicken houses were made on semi-intensive holdings to directly assess some of the biosecurity measures in place.

Finally, animal health services were discussed. This included both traditional and formal veterinary services. Traditional services included those based on indigenous ethnoveterinary knowledge, whereas formal services included biomedically trained veterinarians, animal health assistants and animal health technicians. Additional information was sought regarding the use of other services, such as (human) pharmacies. Discussion included the range of services available and constraints to accessing veterinary services. Initially, these constraints were identified and listed, and subsequently compared, using proportional piling (backyard producers) or ranking (semi-intensive producers).

### 2.5 Data analysis

Data arising from each exercise, including key points from discussions, were recorded in a field notebook and the results of all exercises creating visual representations of data were captured on a digital camera and later transferred to the notebook. Data obtained were qualitative and semi-quantitative in nature and were stored in a spreadsheet programme (Microsoft Excel 2010 Microsoft Cooperation, USA). Data analyses and plotting were performed using R (http://www.R-project.org/).

## 3 Results

A total of 71 poultry keepers participated in this study, including 41 backyard chicken keepers, who took part in 8 focus groups, and 30 semi-intensive chicken producers, who participated in individual interviews. The participants (30 male and 41 female) all agreed that chicken production was particularly important for women. However, it was noted that, in the main, women manage small backyard flocks whereas men often control larger semi-intensive flocks.

**Fig. 1:**
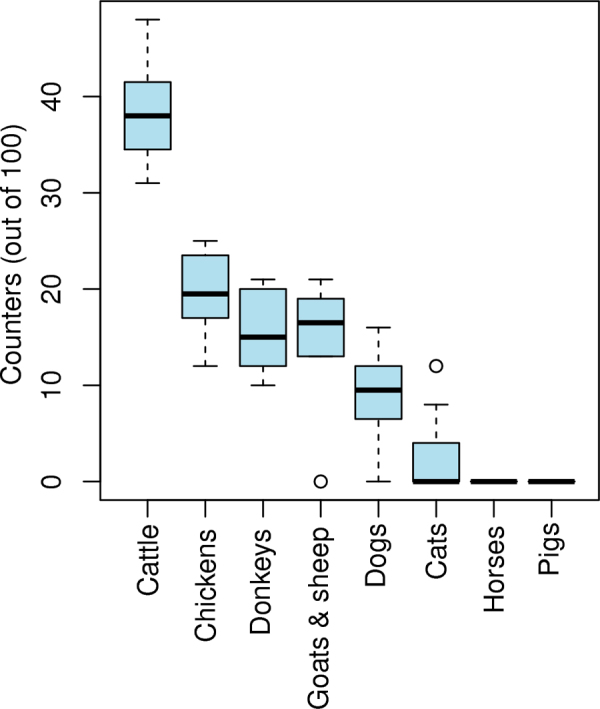
Economic importance of livestock species to backyard chicken farmers (*n* = 8 focus groups) in and around Debre Zeit, Ethiopia. Farmers first identified important species during group discussion then estimated the relative economic importance of each species by allocating an appropriate proportion of 100 counters to each species, with more counters indicating greater importance.

Although detailed information was not recorded for each individual during discussion of livelihood activities, it was noted that almost all participants reported that they were engaged, to some extent, in mixed livelihood activities; these included a variable mix of agricultural (both crop and livestock) and non-agricultural activities (such as trading and civil service). Only three people (all semi-intensive chicken producers) reported that chicken production was their sole source of income. Among the livestock activities, cattle ranked as the most economically important animal to backyard chicken producers in all groups, receiving a median of 38 of the 100 counters (Fig. 1). Chickens (median 19.5 counters) donkeys (16.5) and sheep and goats (15) were the next most economically important animals, although there was wide variation between the groups as to the order in which they ranked these species. Dogs and cats were given low rankings, but were valued as guard animals and for rodent control, respectively. Backyard producers did not mention pigs or horses as economically important species.

Assessment of the economic importance of other animal species was not undertaken with the three semi-intensive chicken farmers who reported chicken production to be their sole source of income. The majority of the remaining semi-intensive chicken farmers (23/27; 74%) ranked chickens as the most (15/27; 56%) or second-most (8/27; 30%) economically important species (Table 1); although for 4 semi-intensive farmers, chickens were not among the top two livestock species in terms of economic importance. Twelve semi-intensive chicken farmers ranked cattle as the most important species. Ten farmers attributed some economic importance to donkeys and seven attributed importance to goats and sheep. Fifteen farmers recognised an economic importance for dogs and three for cats. Thirteen farmers identified an economic importance of horses, albeit usually these animals received a low rank. Only one farmer reported pigs to be economically important. Many participants also engaged in other agricultural (such as crop production) and (particularly semi-intensive producers) non-agricultural activities such as trade, and office and other salaried work.

In all, participants identified 9 constraints to production; seven of 8 groups of backyard producers ranked disease as the most important constraint to chicken production, and another group ranked disease second. Disease was also considered important by many semi-intensive farmers, being identified as a constraint by 15/30 producers (Table 2). Although individuals or individual focus-groups could typically only identify and describe a few diseases, taken in total the participants were able to name and provide accurate clinical signs for at least 9 diseases and syndromes including: Newcastle disease (ND; 22/30 semi-intensive producers and 7/8 backyard producer focus groups); diarrhoea (8/30 and 3/8); chronic respiratory disease (CRD; 2/30 and 5/8); pasteurellosis (1/30 and 2/8); and fowl pox (2/30 and 2/8). Only semi-intensive producers named coccidiosis, ‘eye disease’ and endoparasites as problems. In addition, further discussion identified that ectoparasitism was commonly observed, but often this was not believed to be a cause of morbidity or mortality.

Farmers in this study also demonstrated detailed knowledge of disease risk factors (Table 2). Poor biosecurity and poor management, which included poor feed and housing, were ranked as the most important risk factors by the semi-intensive producers. Backyard producers identified a number of seasonal risk factors, and believed that early morning dew (observed throughout the wet season) was the most important risk factor for disease occurrence. Another seasonal factor included the early onset of the wet season (referred to as *early rain* or *early grass growth*). Alternatively, some farmers simply identified the wet season as increasing risk. Potential routes of contact with infected chickens were also identified, including the scavenging behaviour of free roaming chickens and chicken traders moving around with, and selling, sick chickens. A number of producers of both types identified dogs bringing infected carcasses home to be a risk factor for disease. Despite this, 11/30 semi-intensive producers and all backyard groups reported throwing carcasses away outside the home compound, and a further five semi-intensive producers reported feeding carcasses to dogs.

**Fig. 2:**
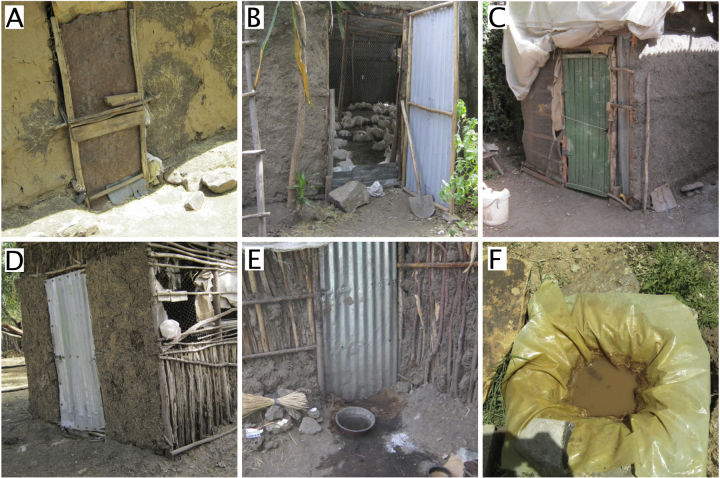
Examples of chicken housing on semi-intensive holdings in and around Debre Zeit, Ethiopia. Materials used in construction made cleaning and disinfection impractical. Footbaths were only used on 2 of the 30 semi-intensive production premises (pictures E and F) and in both cases were unlikely to be effective.

Biosecurity measures on most of the farms visited were lacking or likely to be ineffective. Reasons given for the relative lack of biosecurity included both practical concerns (such as the high cost of disinfectants) and beliefs about disease (such as the perception that biosecurity was only important when chicks were young and more vulnerable to infection). Cleaning of chicken houses usually included manual removal of manure and bedding, which was subsequently used (or sold) as fertiliser. Additional cleaning and disinfection was uncommon and only 2 of the 30 semi-intensive farms visited had a footbath; however these footbaths were unlikely to be effective on the day observed as the solution in one was mostly water and the other contained high levels of organic material (Fig. 2). Even when cleaning and disinfection of houses was reported, the materials used to build the poultry houses (wood, mud and cow dung) made it difficult or impossible for adequate cleaning. Although efforts were sometimes made to exclude rodents and wild birds (such as by using wire mesh on windows), even when present these were observed to be inadequate to prevent access by these animals.

During the ranking exercise, inadequate veterinary services was reported to be a constraint by 9/30 semi-intensive chicken producers and by just 1/8 backyard producer groups. However, even where this was not identified as a constraint per se, 16/30 semi-intensive producers and all backyard producer groups identified one or more issues with local veterinary services and subsequent discussion revealed very limited use of veterinary services for chicken health issues by many respondents. Among the semi-intensive producers the most commonly reported issues included cost, lack of accessibility/availability of veterinary services and lack of the necessary expertise among these service providers, but even so, these were each identified by less than one-third of semi-intensive producers. These three factors were also those most commonly identified by backyard producers, but only cost and accessibility were identified by more than half the groups. Several producers and groups identified that small flock size inhibited access to veterinary services, due to the lower cost-effectiveness of such services. Overall, backyard producers reported little contact with the veterinary sector or knowledge of the services they could provide.

Four semi-intensive producers and 2 backyard groups reported that poultry vaccines were only intermittently available. Further discussion highlighted that availability of vaccines only in inappropriately large volumes was also recognised as an issue by both types of producers. Of respondents who used vaccines on their farms, over 80% administered them themselves, using varying schedules and dilutions contrary to the manufacturer's recommendations. Key informants interviewed during the course of the study corroborated this finding.

The administration of tetracyclines (accessed directly from pharmacies) to chickens was reported in all the backyard producer focus groups, and by 7/30 semi-intensive producers. None of the respondents who reported using tetracycline were aware of appropriate dose rates and many reported adjusting the dose according to their perception of the severity of the illness. The use of other human antibiotic preparations, including amoxicillin, was also mentioned.

Common ethno-veterinary treatments identified by respondents included the use of a plant locally called ‘melia’ (*Melia azedarach*), which was used for a wide range poultry diseases, and pepper and garlic which were used to treat respiratory infections.

The poor quality and cost of feed was an important production constraint to farmers in this study, especially to semi-intensive producers with 25/30 identifying this factor as a constraint, and 10 and 9 ranking it as the most or second-most important constraint, respectively. In contrast, 5/8 backyard producer groups identified feed as a constraint, but only one group ranked it as the most important constraint.

Another constraint highly ranked by semi-intensive farmers was the cost and intermittent availability of chicks, due to what some participants referred to as an ‘absolute monopoly’ of the supply of day-old-chicks by the few active importers and hatcheries. Many semi-intensive producers reported having to wait for a minimum of 4–6 months, and sometimes up to a year, before ordered chicks were supplied, despite, in some cases, up-front payment. In addition, day-old-chicks were often supplied to a large number of producers within a short period of time (i.e. weeks or months of each other) potentially resulting in oversupply at the time of sale leading to reduced market prices. This may compound the effect of poor markets, which was identified as an important constraint by some semi-intensive producers.

Several factors associated with housing were also identified as constraints, particularly by backyard producers. These included provision of shelter, prevention of predation and disputes with neighbours, the latter due to destruction of crops by scavenging chickens. Some backyard producers identified neighbour conflict as a factor limiting the flock size kept by the household, especially during the crop season.

## 4 Discussion

This study investigated the views of people involved in backyard and semi-intensive chicken production in and around Debre Zeit, Ethiopia, with respect to the economic importance of chickens, constraints to production, perceptions of disease risk factors and biosecurity measures. As identified in previous studies (Bradley, 1992; Guèye, 1998; Branckaert and Guèye, 1999; Bravo-Baumann, 2000; Pederson et al., 2001; Seeberg, 2002; Riise et al., 2005; Aklilu et al., 2007; Halima et al., 2007), there was consensus among participants that chicken production was particularly important for women.

For many of the participants in this study chicken production, although important, was not their primary source of income. This is common in Ethiopia where mixed livelihoods predominate (Bluffstone et al., 2008; Huluka et al., 2010). Although detailed data on participants’ income or assets were not collected we believe that it is likely that many (particularly, but not only the backyard producers) had low incomes and little disposable cash, based on our discussions with the participants, our observations during visits to households and our knowledge of the local area. Given these factors, it may not be surprising for most producers to make relatively limited investment in chicken production.

Many of the participants in this study gained economic benefit from a range of livestock and other animal species. These benefits occurred due to both direct effects (such as from production of cattle, sheep and goats) and indirect benefits, such as guard animals (dogs) and rodent control (cats). The role of horses and donkeys was not assessed in detail, but can arise from income-generating activities and/or through their role in transportation. It was not surprising that few people in this study identified pigs as an economically important species. Despite some recent increases, pig production remains low in Ethiopia, where the main religions (Ethiopian Orthodox Christianity and Islam) prohibit consumption of pork.

Taken together, knowledge of chicken diseases among the participants on this study was not poor, and collectively they were able to name and provide accurate clinical signs for numerous diseases and syndromes. This is contrary to previous reports (Pagani and Wossene, 2008), and our own unpublished data from other areas of Ethiopia, where individually and collectively chicken farmers were unable to identify more than a small number of diseases. This difference may be due to the relatively greater importance of chicken production (FAO, 2008; USAID, 2010) and the concentration of animal health expertise in the study area. Many previous studies have focused on Newcastle disease as an important cause of mortality among chickens in Ethiopia (Dessie and Jobre, 2004; Tadesse et al., 2005; Halima et al., 2007) and this is consistent with the opinions of the participants of this study. Newcastle disease was the most frequently identified disease problem causing bird mortality for both types of farmers, and was usually the highest ranked disease. However, our results also highlight that both semi-intensive and backyard farmers believe that a number of other non-specific syndromes and specific pathogens are impacting chicken productivity and mortality, including diarrhoea, chronic respiratory diseases, pasteurellosis and fowl pox. Some potentially significant diseases, such as Marek's disease and infectious bursal disease, were not mentioned, although outbreaks of these are known to have occurred on commercial farms in Ethiopia, including in Debre Zeit, in recent years (Zeleke et al., 2002; Lobago and Woldemeskel, 2004; Jenbreie et al., 2012). This may highlight a lack of knowledge of these important pathogens among the backyard and semi-intensive producers.

Farmers in this study demonstrated detailed knowledge of disease risk factors. Despite recognition by some respondents that dogs bringing infected carcasses home could be a risk factor for disease, 90% of respondents reported disposing of carcasses by throwing them away outside the home compound. Given the potential for pathogen transmission via this route, we advocate identification and promotion of other locally appropriate methods of carcass disposal, preferably through further participatory action research. It is noteworthy that all disease risk factors identified by the participants in this study are consistent with a theory of natural (rather than supernatural) causation of illness (Green, 1999). Due to the urban and peri-urban location of the participants of this study, the extent to which this reflects a more general understanding of (animal) disease causation in Ethiopia is unknown. It should also be noted, however, that detailed exploration of local theories of causation of disease was not undertaken in this study and hence still requires further evaluation.

A key finding of this study was the limited access to veterinary services reported by both semi-intensive and backyard producers. Predominantly, this issue emerged through the informal discussions that followed the ranking exercise about veterinary services. The issues that were identified included difficulty accessing veterinarians, the cost of their services and the perception that veterinarians and other animal-health providers lacked expertise in chicken health and production. Access to veterinary services may be a greater problem for those with smaller flocks, as these producers may perceive engaging with animal health professionals to be less cost-effective and they may also be less attractive clients to the service providers. There was evidence that producers had limited knowledge of the potential benefits of veterinary services, and this may be symptomatic of the previously reported lack of understanding that buyers and sellers of veterinary services in many African countries have of one another (Leonard, 2000). Given the current low interaction between the veterinary profession and chicken producers, efforts to increase chicken health and production through veterinary input may need to include both improved training for veterinarians and efforts to demonstrate the benefits of veterinary input to farmers and veterinarians alike. Animal-health technicians may also be able to make an important contribution to provision of preventive health care.

Poultry vaccines were reported by some respondents to be only intermittently available and then only in inappropriate volumes. Vaccine production (including vaccines against Newcastle disease, infectious bursal disease, fowl pox, fowl typhoid and fowl cholera) was undertaken by a local facility, but this institution faced challenges procuring regular supplies of the raw materials (particularly Specific Pathogen Free eggs) necessary to maintain production. The majority of the vaccines produced by this institution were supplied in 100–200 dose vials, resulting in cost-inflation for farmers with smaller flock sizes, consistent with reports by Moges et al. (2010). However, since this study was conducted, a thermostable Newcastle disease vaccine has become available in 50 dose vials. Evaluation of the impact of this change on vaccine uptake among different farmer groups is warranted. However, it is of concern that, in this study, the majority of respondents who used vaccines did so in ways contrary to the manufacturer's recommendations, highlighting the need for access both to resources and to knowledge. It is worth noting that, due to local production of vaccines, a complex cold chain is not required in this area and transport is not a limiting factor. Hence, this locality provides an opportunity to develop methods to overcome many constraints to vaccine delivery that may be overshadowed in other areas by the technical issues associated with the need for a cold chain and/or enhanced transport infrastructure.

There was widespread use of ethnoveterinary medicine by both groups of producers, although their use appeared somewhat more common among backyard producers. Although this finding was contrary to Pagani and Wossene (2008) who reported limited use of ethno-veterinary medicines in Ethiopia, it is consistent with other studies which have reported the use of ethno-veterinary medicine to control poultry diseases due to lack of funds or access to conventional therapeutics (Guèye, 1997, 1999). One plant commonly reported to be used was *Melia azedarach*, which has demonstrated biological effects, including pesticidal, antiviral, antibacterial and antifungal properties (Al-Rubae, 2009); its use, and that of other ethno-veterinary treatments, deserves more attention and research.

The widespread and potentially inappropriate use of tetracycline reported by participants in this study corroborates previous studies in East Africa (Pagani and Wossene, 2008), and highlights the risks of induction of drug resistance and potential impacts on human health through zoonotic transmission of resistant pathogens, such as *Campylobacter jejuni* (Avrain et al., 2004).

Given that feed constitutes 60–80 per cent of intensive production cost (FAO, 2004) it was not surprising that the poor quality and cost of feed was an important production constraint to farmers in this study, especially for semi-intensive producers. There are few established feed processing companies in Ethiopia and the majority of them are located near Debre Zeit (FAO, 2008). Given that local farmers rank feed constraints so highly, this is a concern for producers in other parts of the country, where the additional transportation costs could be expected to impose further limitations.

Improvement in the supply of day-old-chicks to better match demand from farmers would benefit people engaged in semi-intensive chicken production, although this was not a constraint for backyard producers, who tend to hatch their own stock. However, facilitating this with the private sector suppliers may be difficult due to their own economic goals.

The limited investment in chicken production by most participants in this study almost certainly constrained productivity. However, it would be unfair and, we contend, unhelpful to suggest that improved production would result, in some linear way, from increased expenditure of effort and/or capital by, or on behalf of, the participants, or by Ethiopian chicken producers more generally. Kitalyi (1998) recommended a framework for developing the backyard flock into a semi-commercial venture, starting with the basic indigenous stock, and instituting practices including good hygiene, shelter, preferential treatment of chicks and control of the most devastating diseases, such as Newcastle disease. Later improvements in the management include developing feeding and other disease control programmes, followed by improving the stock by introducing high-yielding traits and developing marketing strategies. In this context, the semi-intensive producers may be thought of as people who, supported by the schemes available from the poultry multiplication centres, have already made investments in poultry but have now run up against a new set of challenges which limit this undertaking. Despite chickens being a valuable asset, the high-risk nature of the enterprise still limits it to being only a part of their livelihood, and it is probable that it is only by having other livelihood activities that they are able to divert capital into improving their poultry production. Backyard producers have yet to make any major commitment to improve their chicken productivity, constrained as they are by a lack of access to some of the most basic healthcare provisions, such as vaccines and veterinary advice. The preference for indigenous poultry products may also mean they are reluctant to subscribe to a scheme based on rearing exotic chickens.

Although women play an important role in backyard production systems, semi-intensive farms are more likely to be controlled by men, and poultry development schemes, which frequently cite one of their aims as empowering women, need to be aware of this. Women are less likely to have access to capital to allow them to make a jump from backyard to semi-intensive producer all at once, and require support to allow them to slowly build up a flock into a profitable venture.

Although backyard and semi-intensive farmers have highlighted different constraints, these partly reflect their differing business models, and common themes can be identified throughout; the overriding ones being difficulties accessing agricultural and veterinary inputs, knowledge and expertise. Many of these constraints could be viewed as simply technical issues to be overcome. However, we believe it is important to recognise the social factors underpinning what are, in reality, relatively modest technical challenges. Veterinary and development service providers need to think of chicken production systems as a continuous spectrum, and recognise and make provision for the different inputs needed for those at each stage of development. This process should occur in partnership with the farmers. In this way, a wide variety of business models can be accommodated, and chicken producers of all kinds can decide how much to invest in production, based on their own requirements and individual situation.

## 5 Conclusion

The study area (Debre Zeit) is a focus of chicken production (FAO, 2008; USAID, 2010) and expertise in Ethiopia. Hence, this area has greater potential for access to production and veterinary expertise and materials than anywhere else in the country. Thus, the results of this study illustrate the constraints to production in close proximity to infrastructure and expertise that could be exploited to support chicken-keepers. Therefore, these results are not representative of most other areas of Ethiopia, where constraints are likely to be more acute, being compounded by infrastructural limitations affecting communications and transportation. Nevertheless, our results suggest that even if technical issues related to communications and transportation were to be overcome elsewhere, the substantial constraints evident in this study would likely remain.

## Figures and Tables

**Table 1:** Economic importance of livestock species to semi-intensive chicken farmers in and around Debre Zeit, Ethiopia (*n* = 27 individuals; note that additional 3 farmers, whose sole source of income was from chicken production, have been excluded). Farmers identified economically important species in individual interviews and then ranked those species from most (rank = 1) to least (rank = 5) important.

	Rank					
	1	2	3	4	5	Unranked
Chickens	15	8	1	2	1	0
Cattle	12	2	1	0	0	12
Donkeys	0	2	7	1	0	17
Goats and sheep	0	1	3	2	1	20
Dogs	0	0	2	7	6	12
Cats	0	2	0	1	0	24
Horses	0	0	2	2	9	14
Pigs	0	1	0	0	0	26

**Table 2:** Important poultry diseases and risk factors, production and veterinary constraints as reported by farmers; ranks 1 = most important, score 5 = least important.

Topic	Variable identified	Semi-intensive producers								Backyard producers							
		Ranking						Median		Ranking						Median	
		1	2	3	4	5	UR	All¶	Ranked§	1	2	3	4	5	UR	All	Ranked
Production constraints	Feed	10	9	5	1	–	5	2	2	1	2	–	1	1	3	4.5	2
	Disease	9	2	2	2	–	15	•	1	7	1	–	–	–	–	1	1
	Day-old chicks	5	4	2	–	–	19	•	2	–	–	–	–	–	8	•	NA
	Poor markets	2	3	3	–	1	21	•	2	–	–	–	–	–	8	•	NA
	Veterinary services	–	4	4	1	–	21	•	3	–	–	–	–	1	7	•	5
	Shelter	2	1	1	–	2	24	•	2.5	–	1	3	1	–	3	3.5	3
	Poor production	0	2	3	–	–	25	•	3	–	1	1	1	–	5	•	3
	Predators	1	2	1	–	–	26	•	2	–	2	3	2	–	1	3	3
	Neighbours	–	–	–	–	–	30	•	NA	–	1	–	2	–	5	•	4

Diseases	Newcastle disease	15	5	–	2	–	8	2	1	7	–	1	–	–	–	1	1
	Diarrhoea	2	4	2	–	–	22	•	2	–	2	1	–	–	5	•	2
	Coccidiosis	2	1	3	–	–	24	•	2.5	–	–	–	–	–	8	•	NA
	Ectoparasitism	0	2	2	–	–	26	•	2.5	–	1	1	1	1	4	5/•†	3.5
	Fowl pox	1	1	1	–	–	27	•	2	–	–	–	1	1	6	•	4.5
	Eye disease	1	1	1	–	–	27	•	2	–	–	–	–	–	8	•	NA
	Chronic resp. disease	–	2	1	–	–	27	•	2	1	2	1	1	–	3	3.5	2
	Pasteurellosis	1	–	–	–	–	29	•	1	–	2	–	–	–	6	•	2
	Endoparasitism	–	1	–	–	–	29	•	2	–	–	–	–	–	8	•	NA

Risk factors	Poor biosecurity	8	8	2	–	–	12	2	2	1	1	–	1	–	5	•	2
	Poor management	8	5	3	1	1	12	3	2	1	–	–	2	1	4	5/•†	4
	Early rain/grass	1	3	3	–	–	23	•	2	–	1	3	1	–	3	3.5	3
	Scavenging	1	2	2	3	–	22	•	3	–	2	2	–	–	4	4.5	2.5
	Dew	3	2	–	–	–	25	•	1	4	1	–	–	–	3	1.5	1
	Wet season	1	3	2	–	–	24	•	2	1	–	1	–	–	6	•	2
	Carcases	2	2	1	–	1	24	•	2	1	3	1	–	1	2	2.5	2
	Trade of sick birds	2	1	2	–	–	25	•	2	–	–	1	1	–	6	•	3.5
	Lack of vaccines	2	–	–	1	–	27	•	1	–	–	–	–	–	8	•	NA

Veterinary services	Cost	6	3	–	–	–	21	•	1	1	4	–	1	–	2	2	2
	Vet accessibility	4	2	2	–	–	22	•	1.5	5	–	–	–	–	3	1	1
	Lack of expert vets	2	2	2	–	–	24	•	2	1	2	1	–	–	4	3/•†	2
	Lack of vaccines	1	3	–	–	–	26	•	2	–	–	1	–	1	6	•	4
	Ineffective drugs	–	2	–	-	–	28	•	2	–	1	1	–	–	6	•	2.5
	Small flock size	1	–	1	–	–	28	•	2	–	–	1	–	–	7	•	3
	Lack of drugs	–	–	–	–	–	30	•	NA	1	–	–	–	–	7	•	1

UR – unranked. NA – not applicable, as not mentioned (and, therefore, ranked) by any individuals/groups. • Not mentioned (or, therefore, ranked) by at least half of all respondents/groups.

¶ Calculated using all responses, including individuals or groups that did not identify (and therefore did not rank) an item.

§ Median of ranked responses; individuals or groups that did not identify (and therefore did not rank) an item were excluded.

† Median value falls between lowest ranked value and ‘unranked’, as exactly half the groups did not rank the item.

## References

[bib0005] Abdelqader A., Wollny C.B., Gauly M. (2007). Characterization of local chicken production systems and their potential under different levels of management practice in Jordan. Trop. Anim. Health Prod..

[bib0010] Abubakar M.B., Ambali A.G., Tamjdo T. (2007). Rural chicken production: effects of gender on ownership, and management responsibilities in some parts of Nigeria and Cameroon. Int. J. Poult. Sci..

[bib0015] Adesehinwa A.O.K., Okunola J.O., Adewumi M.K. (2003). Socio-economic characteristics of ruminant livestock farmers and their production constraints in some parts of South-western Nigeria. Livest. Res. Rural Dev..

[bib0020] Admassu B., Sones K., Catley A. (2003). Primary animal healthcare in Ethiopia: the experience so far. Primary Animal Healthcare in the 21st Century: Shaping the Rules, Policies and Institutions.

[bib0025] Aklilu H.A., Almekinders C.J.M., Van der Zijpp A.J. (2007). Village poultry consumption and marketing in relation to gender, religious festivals and market access. Trop. Anim. Health Prod..

[bib0030] Al-Rubae A.Y. (2009). The potential use of *Melia azedarach* L. as pesticidal and medicinal plant, review. Am.-Eurasian J. Sustain. Agric..

[bib0035] Alemu D., Degefe T., Ferede S., Nzietcheung S., Roy D. (2008). Overview and Background Paper on Ethiopia's Poultry Sector: Relevance for HPAI Research in Ethiopia. DFID Pro-poor HPAI Risk Reduction Strategies Project Africa/Indonesia Region Report No. 1.

[bib0040] Avrain L., Vernozy-Rozand C., Kempf I. (2004). Evidence for natural horizontal transfer of tetO gene between *Campylobacter jejuni* strains in chickens. J. Appl. Microbiol..

[bib0045] Ayele G., Asare-Marfo D., Birol E., Roy D. (2009). Investigating the Role of Poultry in Livelihoods and the Impact of HPAI in Ethiopia. Controlling Avian Flu and Protecting People's Livelihoods in Africa and Indonesia. International Food Policy Research Institute (IFPRI) with the International Livestock Research Institute (ILRI) and Royal Veterinary College (RVC).

[bib0050] Bluffstone R., Yesuf M., Bushie B., Damite D. (2008). Rural Livelihoods, Poverty, and the Millennium Development Goals: Evidence from Ethiopian Survey Data. Environment for Development Discussion Paper Series, EfD DP 08-07. http://www.rff.org/RFF/Documents/EfD-DP-08-07.pdf.

[bib0055] Bradley F.A. (1992). A historical review of women's contributions to poultry production and the implications for poultry development process. Proceedings of the 19th World's Poultry Congress.

[bib0060] Branckaert R.D.S., Guèye E.F., Dolberg F., Petersen P.H. (1999). FAO's programme for support to family poultry production. Poultry as a Tool in Poverty Eradication and Promotion of Gender Equality.

[bib0065] Bravo-Baumann H. (2000). Capitalisation of experiences on the contribution of livestock projects and gender. Livestock and Gender: A Winning Pair.

[bib0070] CACC (2003). http://www.csa.gov.et/newcsaweb/images/documents/surveys/Livestock/Livestock_2003_2004/survey0/data/docs/pdf/report/LV2004F.pdf.

[bib0075] Catley A., Mariner J. (2001). Participatory Epidemiology: lessons learned and future directions. Proceeding of a workshop held in Addis Ababa.

[bib0080] CSA (2005). Agricultural Sample Survey 2004/05, Volume II; Report on Livestock and Livestock Characteristics (Private Peasant Holdings). Central Statistical Authority, Statistical Bulletin, Addis Ababa, Ethiopia.

[bib0085] CSA (2010/11). Report on Livestock and Livestock Characteristics (Private Peasant Holdings).

[bib0090] Dana N., van der Waaij L.H., Dessie T., van Arendonk J.A. (2010). Production objectives and trait preferences of village poultry producers of Ethiopia: implications for designing breeding schemes utilizing indigenous chicken genetic resources. Trop. Anim. Health Prod..

[bib0095] Demeke S. (1996). Study on egg production of white leghorn under intensive, semi-intensive and rural household conditions in Ethiopia. Livest. Res. Rural Dev..

[bib0100] Dessie T., Jobre Y. (2004). A review of the importance and control of Newcastle disease in Ethiopia. Ethiop. Vet. J..

[bib0105] Dinka H., Chala R., Dawo F., Bekana E., Leta S. (2010). Major constraints and health management of village poultry production in Rift Valley of Oromia, Ethiopia. Am.-Eurasian J. Agric. Environ. Sci..

[bib0110] FAO (1997). Guidelines for the Inclusion of Improved Household Poultry Production. Special Programme for Food Security.

[bib0115] FAO (2004). Livestock Sector Brief: Ethiopia. http://www.fao.org/ag/againfo/resources/en/publications/sector_briefs/lsb_ETH.pdf.

[bib0120] FAO (2008). Poultry Sector Country Review. http://www.fao-ectad-nairobi.org/IMG/pdf/Ethiopia_Poultry_Sector_Review.pdf.

[bib0125] Gondwe T.N.P. (2004). Characterization of Local Chicken in Low Input–Low Output Production Systems: Is There Scope for Appropriate Production and Breeding Strategies in Malawi?.

[bib0130] Green E.C. (1999). Indigenous Theories of Contagious Disease.

[bib0135] Guèye E.F. (1997). Diseases in village chickens: control through ethno-veterinary medicine. ILEIA Newsl..

[bib0140] Guèye E.F. (1998). Village egg and fowl meat production in Africa. World Poult. Sci. J..

[bib0145] Guèye E.F. (1999). Ethno veterinary medicine against poultry diseases in African villages. World Poult. Sci. J..

[bib0150] Guèye E.F. (2003). Gender issues in family poultry production systems in low-income food-deficit countries. Am. J. Altern. Agric..

[bib0155] Guèye E.F., Bessei W. (1996). Geflügelhaltung in Afrika: Bedeutung und Perspektiven. Gefliigelwirtschaft Schweineprod. Mag..

[bib0160] Halima H., Neser F.W.C., Marle-Koster E., Kock A. (2007). Village-based indigenous chicken production system in north-west Ethiopia. Trop. Anim. Health Prod..

[bib0165] Huluka T.A., Hamda H., Tesfaye T. (2010). Livelihood Strategies of the Rural Poor in Ethiopia: Households Poverty, Livelihood Strategies and Factors Affecting Livelihood Choice in Rural Ethiopia.

[bib0170] Jenbreie S., Ayelet G., Gelaye E., Kebede F., Lynch S.E., Negussie H. (2012). Infectious bursal disease: seroprevalence and associated risk factors in major poultry rearing areas of Ethiopia. Trop. Anim. Health Prod..

[bib0175] Kitalyi A.J. (1998). Village Chicken Production Systems in Rural Africa Household Food Security and Gender Issues.

[bib0180] Leonard D.K., Leonard D.K. (2000). The new institutional economics and the restructuring of animal health services in Africa. Africa's Changing Market for Health and Veterinary Services.

[bib0185] Lobago F., Woldemeskel M. (2004). An outbreak of Marek's disease in chickens in Central Ethiopia. Trop. Anim. Health Prod..

[bib0190] Mariner J.C., Paskin R. (2000). FAO Animal Health Manual 10 – Manual on Participatory Epidemiology – Method for the Collection of Action-oriented Epidemiological Intelligence.

[bib0195] Mazengia H. (2012). Review on major viral diseases of chickens reported in Ethiopia. J. Infect. Dis. Immunity.

[bib0200] Moges F., Tegegne A., Dessie T. (2010). Indigenous Chicken Production and Marketing Systems in Ethiopia: Characteristics and Opportunities for Market-oriented Development. IPMS (Improving Productivity and Market Success) of Ethiopian Farmers Project Working Paper 24. http://results.waterandfood.org/bitstream/handle/10568/2685/WorkingPaper_24.pdf%3Fsequence=4.

[bib0205] Pagani P., Wossene A. (2008). Review of the New Features of the Ethiopian Poultry Sector Biosecurity Implications.

[bib0210] Pederson C.V., Kristensen A.R., Madsen J. (2001). On-farm research leading to a dynamic model of traditional chicken production systems. Proceedings of the Joint 17th Scientific Conference of the Tanzania Society for Animal Production and the 20th Scientific Conference of the Tanzania Veterinary Association.

[bib0215] Riise J.C., Permin A., Kryger K.N. (2005). Strategies for developing family poultry production at village level – experiences from West Africa and Asia. World Poult. Sci. J.

[bib0220] Seeberg D.S. (2002). Money in My Hand: An Anthropological Analysis of the Empowerment of Women Through Participation in Participatory Livestock Development Project in Northwest Bangladesh. Field Report Based on Ethnographic Fieldwork in Bangladesh.

[bib0225] Sonaiya E.B. (1990). The context and prospects for development of small holder rural poultry production in Africa. Proceedings of the CTA Seminar on Small holder Rural Poultry Production.

[bib0230] Sonaiya E.B., Mack S. (1990). Towards sustainable poultry production in Africa. Proceedings of the FAO Expert Consultation.

[bib0235] Tadelle D., Million T., Alemu Y., Peters K.J. (2003). Village chicken production systems in Ethiopia: 2. Use patterns and performance valuation and chicken products and socio-economic functions of chicken. Livest. Res. Rural Dev..

[bib0240] Dessie T., Ogle B. (2001). Village poultry production systems in the central highlands of Ethiopia. Trop. Anim. Health Prod..

[bib0245] Tadesse S., Aschenafi H., Aschalew Z. (2005). Seroprevalence study of Newcastle disease in local chickens in Central Ethiopia. Int. J. Appl. Res. Vet. Med..

[bib0250] USAID (2010). Partnership for Safe Poultry in Kenya (PSPK) Program: Value Chain Analysis of Poultry in Ethiopia. http://pdf.usaid.gov/pdf_docs/PNADU076.pdf.

[bib0255] Wilson R.T. (2010). Poultry production and performance in the Federal Democratic Republic of Ethiopia. World Poult. Sci. J..

[bib0260] Wolde S., Negesse T., Melesse A. (2011). The effect of dietary protein concentration on nutrient utilization of Rhode Island red chicken in Wolaita (Southern Ethiopia). Trop. Subtrop. Agroecosyst..

[bib0265] Woldemariam S., Wossene A. (2007). Infectious bursal disease (Gumboro Disease): case report at Andasa poultry farm, Amhara region. Ethiop. Vet. J..

[bib0270] Wossene A. (2006). Poultry Bio-security Study in Ethiopia. A Consultancy Report to the Food and Agricultural Organisation (FAO), Addis Ababa, Ethiopia.

[bib0275] Zeleke A., Yami M., Kebede F., Melese N., Senait B., Gelaye E., Sori T. (2002). Gumboro: an emerging disease threat to poultry farms in Debre Zeit. Ethiop. Vet. J..

